# Challenges, opportunities and best practices in routine childhood vaccination services delivery in urban slums and pastoralist regions of Ethiopia: An exploratory qualitative study in marginalized settings

**DOI:** 10.1371/journal.pone.0356253

**Published:** 2026-08-21

**Authors:** Nardos Gelana, Finina Abebe, Menen Tsegaw, Yordanos Tadesse, Melkamu Ayalew Kokebie, Tseganesh Gedlu, Hanna Alemayehu, Tesfaye Bikes, Yeshiwork Eshetu, Mesfin Kassaye, Adugna Endale, Rachana Sharma, Hiwot Getachew, Hnin Su Mon, Tolasa Yadate

**Affiliations:** 1 Ethiopian Health Education and Promotion Professionals Association (EHEPA), Addis Ababa, Ethiopia; 2 Clinton Health Access Initiative (CHAI), Addis Ababa, Ethiopia; 3 Department of Public Health, College of Medicine and Health Sciences, Ambo University, Ambo, Ethiopia; 4 School of Public Health, Addis Ababa University, Addis Ababa, Ethiopia; 5 Immunization Service Desk Lead, Ethiopia Ministry of Health, Addis Ababa, Ethiopia; 6 Immunization Service Desk Consultant, Ethiopia Ministry of Health, Addis Ababa, Ethiopia; 7 Immunization Service Desk, National Communication and Demand generation TA, Ethiopia Ministry of Health, Addis Ababa, Ethiopia; 8 Immunization Service Desk, SBCC Advisor, Ethiopia Ministry of Health, Addis Ababa, Ethiopia; 9 Immunization Service Desk, Immunization Service expert, Ethiopia Ministry of Health, Addis Ababa, Ethiopia; 10 Immunization Service Desk, National Immunization SBC consultant, Ethiopia Ministry of Health, Addis Ababa, Ethiopia; 11 National Consultant for Tailored Immunization Strategies, UNICEF, Addis Ababa, Ethiopia; 12 United Nations Children’s Fund (UNICEF), Addis Ababa, Ethiopia; 13 School of Public Health, College of Health Sciences and Medicine, Dilla University, Dilla, Ethiopia; PLOS: Public Library of Science, ETHIOPIA

## Abstract

**Background:**

Despite global progress, significant disparities persist in routine childhood vaccination coverage, commonly in low- and middle-income countries, including Ethiopia. Ethiopia has made considerable progress in extending immunization services through the national Expanded Program on Immunization. However, still significant coverage gaps remain, particularly among the vulnerable population such as urban slums and pastoralist areas. This study seeks to capture the perspectives of healthcare professionals and program managers to understand the challenges, best practices, and opportunities for strengthening immunization service delivery in these marginalized settings.

**Methods:**

An exploratory qualitative study was conducted in urban slum and pastoralist areas of Ethiopian regions from November to December, 2023. Purposive sampling technique was used to select 39 key informants, including healthcare providers, health extension workers, maternal and child health directors, Woreda Immunization officers, and focal persons. The interview guides were developed with Human-Centered Designand the World Health Organization’s behavioral and social drivers frameworks. Open Code software version 4.03 was used to perform thematic analysis, employing both deductive and inductive coding approaches. Inter-coder reliability and daily debriefing were used to ensure the trustworthiness of the study.

**Results:**

Key barriers affecting the routine childhood immunization service included inconsistent vaccine supply, limited cold-chain capacity, low financing, high workloads, and challenges in reaching these populations. However, best practices such as community engagement, involvement of local community structures, mobile teams and the inclusion of the private sector demonstrated notable successes. Healthcare professionals recommended improving supply chains, enhancing healthcare providers training and supervision, and increasing community awareness as crucial strategies to reach under-immunized groups.

**Conclusion:**

This study identified that persistent health system challenges remain in delivery of routine childhood vaccination, while there is best practices in some areas. Strengthening health system capacity in terms of human power and necessary, combined with active community engagement, is essential to achieving equitable vaccine coverage among under-immunized populations.

## Introduction

Immunization remains one of the most productive and cost-effective public health interventions, globally preventing more than 30 life-threatening diseases and infections and an estimated 3.5 to 5 million deaths annually from vaccine-preventable diseases [1,2]. Despite global progress, significant disparities persist in routine childhood vaccination coverage, commonly in low- and middle-income countries (LMICs), including Ethiopia. Geographic, socioeconomic, and systemic challenges continue to hinder equitable access to immunization services, especially among those living in urban slums and pastoralist regions [3,4].

Ethiopia has made considerable progresses in extending immunization services through the national Expanded Program on Immunization (EPI). However, still significant coverage gaps remain, particularly among the vulnerable populations. The 2019 Ethiopian Mini Demographic and Health Survey revealed regional differences in immunization rates, with children in remote and marginalized areas being under-vaccinated [5]. This inequity is worsened by unique challenges to these contexts, including high mobility in pastoralist community, poor infrastructure, limited health workforce capacity, and socio-cultural barriers such as low health literacy and vaccine hesitancy [4,6,7].

Urban slum areas are characterized by different yet equally complex set of challenges. Rapid urbanization has led to the expansion of informal settlements where public services, including healthcare, are less accessible or absent at all in those areas [8–10]. These environments often suffer from high population density and limited health systems, which contribute to suboptimal vaccine uptake [11]. Similarly, the mobility and hard to reach situation of pastoralist communities lead to logistical and operational barriers to accessing routine immunization services, such as limited cold chain facilities and inconsistent outreach services by health providers [12].

Health system factors including logistics and supply chain, healthcare worker motivation and training, managerial commitment, and community involvement play a crucial role in affecting the delivery and uptake of vaccination services [13,14]. Understanding how these factors are interconnected in marginalized settings is essential for designing effective and tailored interventions

Although immunization coverage and associated barriers have been well documented by several quantitative studies, the lack of in-depth qualitative research exploring the insights of frontline health workers and stakeholders, particularly among underserved communities, remains limited. Interventions based on behavioral and social research tend to be more cost-effective; furthermore, they tend to lead to better results than interventions that are not informed by behavioral science research, because they target barriers identified with the communities’ context, needs and expectations [15]. Hence, the Behavioral and Social Drivers (BeSD) framework for vaccination was applied during the implementation of this research [16].

This study aimed to fill the evidence gap by exploring the health system barriers and facilitators influencing the delivery of routine childhood vaccination services in urban slum and pastoralist regions of Ethiopia specifically in five regions including Addis Ababa, Oromia, Gambella, Afar and Somali regions. This study seeks to capture the perspectives of healthcare professionals and program managers to understand the challenges, best practices, and opportunities for strengthening immunization service delivery in marginalized settings.

The top-down public health approaches fail in these contexts because they do not account for the existing realities in these marginalized settings. To design truly inclusive immunization strategies, it needs to change our approach toward empathetic and participatory. Unlike the conventional approaches, Human-Centered Design (HCD) is a powerful framework to deeply understand end-users’ perspectives and co-create tailored solutions [17–20]. Therefore, this qualitative study aims to identify barriers, opportunities and best practices of routine immunization service delivery using HCD approachto inform the development of tailored strategies to promote vaccination uptake and address equity gaps in Ethiopian urban-slums and pastoralist settings.

## Materials and methods

### Study approach and context

A qualitative exploratory study was conducted in urban slum and pastoral areas of Ethiopia, specifically in Addis Ababa, Oromia, Gambella, Afar, and Somali regions, from November to December 2023. Those regions were selected based on their number of zero dose under five children. In addition the study included pastoralist regions (Gambella, Somali and Afar) due to high number of zero-dose and under-immunised children. The selection was done in consultation with UNICEF Ethiopia and Ministry of Health Ethiopia.

### Participant selection

Healthcare providers, HEWs, RMNCH directors, Woreda EPI officers, and EPI focal persons from selected health centers who were engaged in the immunization program were included in the study. Purposive sampling was used to gather in-depth information about immunization services. Key informants were chosen from Regional Health Bureau, RMNCH directors, Woreda EPI officers, and EPI focal persons from selected health centers.Criterion-based purposive sampling was employed to recruit participants who met predefined inclusion criteria. Eligible participants were individuals with direct experience in the immunization program both at Health Facility and Woreda Health Offices. In total, 39 key informant interviews were conducted, including 5 Reproductive, maternal, newborn and Child Health (RMNCH) directors, 5 Woreda EPI officers, 5 EPI focal persons, 14 healthcare workers (HCW), and 10 health extension workers (HEW). The final sample size was determined based on the concept of idea saturation whereby participants were purposely selected based on their extensive experience and expertise. Interviews continued until sufficient depth and breadth of information had been obtained and no new themes emerged during ongoing analysis.

### Data Collection

Data was collected using key informant interview (KII) guides. The interview guide was developed after reviewing relevant literature and utilizing HCD and BeSD frameworks for vaccination [16,17]. The study tools were translated into local languages, and all consent forms were provided in Amharic, Afan Oromo, Somali, Afar, and Nuer languages.The interview guides were translated from English into the respective local languages by bilingual professionals familiar with the local context and public health terminology. To ensure linguistic accuracy and conceptual consistency, the translated versions were reviewed by members of the research team and local language experts. A validation workshop was held with stakeholders and the national Immunization communication technical working group in Addis Ababa. The workshop participants were experts who have been working on immunization programs and projects. Following the workshop, the study tools were revised based on the experts feedback to enhance content, clarity, cultural acceptability, and relevance prior to data collection.

### Data analysis

The audio recordings from the key informant interviews were documented and translated into English. Field notes were taken during the interviews. All interview transcripts were reviewed multiple times to ensure a comprehensive understanding of the dataset. The translated data was coded using Open Code 4.03 software. Coding was conducted iteratively by the research team. Throughout the analysis, regular meetings were held to compare coding decisions, discuss emerging concepts, and resolve differences in interpretation. Consensus was achieved through discussion, and the coding framework was continuously refined as new insights emerged, thereby enhancing the credibility and trustworthiness of the findings. The coding process combined both deductive and inductive methods: preliminary codes and themes were defined deductively based on the research questions, while additional codes that emerged during analysis were incorporated inductively. The investigators created codes and organized them into broader themes. Once the data coding was finalized, thematic analysis was performed. The preliminary results were reviewed by the research team, and consensus was reached to ensure alignment with the research objectives and scientific validity. Ultimately, the findings were presented in narrative form, including relevant quotes to support the identified themes.

### Trustworthiness

To ensure the trustworthiness of the study, quality assurance measures were implemented throughout the research process. Prior to data collection, the interview guide was validated with relevant stakeholders, and revisions were made based on their feedback to enhance its clarity and relevance. Data collectors, field facilitators, and supervisors received comprehensive training to ensure a shared understanding of the study objectives, interview procedures, and ethical considerations, thereby promoting the credibility and dependability of the data collection process.

During data collection, supportive supervision, daily debriefing sessions, and regular communication with data collectors were conducted to monitor data quality, address emerging issues, and ensure the consistent application of the study procedures, thereby strengthening credibility and dependability. During data analysis, transcribed interviews were reviewed against the original interview records to verify the accuracy and completeness of the transcripts and to ensure that the findings faithfully reflected participants’ perspectives, thereby enhancing confirmability. Transferability was supported through the purposeful selection of information-rich participants who met predefined inclusion criteria and by providing detailed descriptions of the study context and participant characteristics, enabling readers to assess the applicability of the findings to similar settings. In addition, regular discussions and feedback among the research team throughout data collection and analysis contributed to the credibility and confirmability of the study findings.

### Ethical approval and consent to participate

Ethical clearance was obtained from the Ethiopian Public Health Association (EPHA) Institutional Review Board (reference number: EPHA/OG/902/23). A support letter was obtained from the Ministry of Health (MOH) and the respective regional and city administration health offices. After the purpose and objective of the study were informed, written informed consent was obtained from each study participant. All participants were informed that participation was on a voluntary basis and they could withdraw from the study at any time if they were not comfortable with the questions. The data were collected and analyzed anonymously by removing personal identifiers from the data in order to maintain confidentiality.

### Findings

#### Study participants profile.

A total of 39 participants were purposively recruited from five regions of Ethiopia (Oromia, Somali, Afar, Addis Ababa, and Gambella). The study included 24 frontline healthcare providers and 15 key informants involved in the planning, management, and delivery of routine childhood vaccination services. Frontline participants comprised 14 healthcare workers (HCWs), primarily nurses, and 10 health extension workers (HEWs). Key informants included five Reproductive, Maternal, Newborn and Child Health (RMNCH) directors, five woreda EPI officers, and five EPI focal persons representing regional, city, sub-city, and woreda health system levels. Overall, 24 participants were male and 15 were female. The distribution of the study participants are presented in Table 1.

**Table 1:** Distribution of Key Informants and Frontline Healthcare Providers Across Study Regions.

No	Region	Frontline HCWs/HEWs	Key Informants	Total
1.	Oromia	4	3	7
2.	Somali	5	3	8
3.	Afar	6	3	9
4.	Addis Ababa	6	3	9
5.	Gambella	3	3	6
	Total	24	15	39

Barriers affecting the provision of childhood vaccination services in Ethiopian semi-urban and pastoralist communities, enabling conditions and best practices were qualitatively explored based on interviews conducted with key informants. The analysis aimed to identify key themes, sub-themes and codes related to challenges, opportunites and best practices in vaccination service delivery in these settings. Various barriers, enablers and best practices including inconsistent vaccine supply, limited cold-chain capacity, low financing, high workloads, and challenges in reaching these populations and best practices such as community engagement, involvement of local community structures, mobile teams and the inclusion of the private sector. Direct quotes from the interviewees are included to explain these themes and codes.

### Theme one: Knowledge and belief towards vaccination

This theme summarizes the health care providers’ knowledge and beliefs about the childhood routine immunization and the service users. Across regions, most of the healthcare workers believe in the importance of routine immunization for protecting children from vaccine-preventable diseases. The results obtained from the Afar region indicate that a vast majority of healthcare professionals hold the belief that routine immunization is of utmost significance in safeguarding children against various illnesses.

“*Many things can be mentioned about vaccination, but mainly it is used to prevent infectious diseases such as TB, pertussis, measles and it is useful to prevent children before they are affected by diseases*.” *(HEW 2: Female, Afar)*

The belief in the importance of childhood routine immunization remains strong across regions. Participants emphasize the role of immunization in preventing communicable diseases and express the need for early vaccination initiation, starting promptly at birth.

“*When I hear about routine immunization, what comes to my mind is about childcare or benefitting children from communicable diseases that affect them by providing different types of vaccines. Everybody knows what benefits vaccines have, therefore what comes to my mind is how helpful the vaccines are, and that the community is not being benefited as they should be.” (HCW 1: Somali)*

### Theme two: Intention

This theme includes factors that reduce and enhance the intention of health care providers to give routine childhood immunization services. Factors such as low per diem, lack of regular training on updated vaccines, high workloads, inconvenient waiting areas, and transportation issues for outreach activities were identified as barriers in specific regions.

*“I didn’t observe health professionals who were satisfied working at the EPI unit. For example, you will get sad if you come on Wednesday. I can stay there until 5 or 6 pm without eating my lunch. Since only one person is assigned to this unit, everything is covered by a single healthcare provider. You can’t get any support from other departments. I worry about the cold chain because no one gives attention to it, rather than me. To be honest I think that is a barrier to what is going to happen if I am sick, because no health professional observes it. I don’t know why health professionals fear working in EPI clinics.” (HCW 2: Oromia)*

*“The lack of professional training is very difficult and in our health institution only one professional has received training once in the last two years. However, if it is possible to give the vaccination training to the existing health professionals, any other professional can work in place of the vaccinator.” (HEW 3: Female, Afar)*

The pastoral culture of communities, characterized by frequent movement, was also highlighted as a significant barrier affecting the intention of healthcare workers in Afar and Gambella regions.

*“As the afar Community move from place to place there are occasions that we return without getting children because people who were in the area where we were going to provide campaign service have left the area and migrate to another place. When we provide door-to-door vaccination services, the challenge for us is that since the community is pastoralist, they take their animals and go to fetch water or search grazing land so that we aren’t getting children at home. In addition the living condition of the community is very far apart. So the capacity of the vaccinator to reach the entire house will be limited” (HCW 2: Afar)*

Across regions, satisfaction derived from serving children and caregivers found to be a good facilitator for the intention of the healthcare workers. Additionally, factors such as improved vaccine supply, cold chain management, and collaboration with community leaders are mentioned as enablers in multiple regions.

*“We are satisfied because we are helping the children and mothers. Personally, I am so happy working here. I am proud of being health extension worker.” (HEW 1: Oromia)*

The finding from the Somali region also showed that, healthcare workers identified the collaboration with women committees, religious leaders, and kebele leaders in the community as effective enablers for facilitating the vaccination service. Similarly, the findings from the Gambella region identify community participation during vaccine campaigns and client satisfaction as key enabling factors that motivate healthcare workers.

### Theme three: vaccination service experience

This theme summarizes the barriers and facilitators encountered by healthcare providers when delivering routine immunization services, whether at the health facility, through house-to-house visits, or at outreach and mobile sites.

The findings show rumors circulating within the communities, concerning routine immunization, with some caregivers fearing that vaccines will make their children ill. As a result, during outreach programs, some caregivers may refuse to have their children vaccinated due to these concerns.

*“…there was one mother who refused to immunize her child because she had heard someone who died of vaccination adverse event in a place where she used to live previously. So, she refused to immunize her child even though we went to her home and discussed with her several times. She isn’t willing. There could be others who might not be willing to vaccinate their children because of such rumors.” (HCW 1: Addis Ababa)*

Lack of awareness among caregivers about adverse effects of the vaccine, as well as low education levels; pose significant challenges to vaccination services in many regions.

*“The main reason for this issue is the lack of formal education. The main problem are defaulters, due to the reason of the adverse events associated with vaccination. Many children experience side effects like fever and swelling after receiving vaccines, especially the pentavalent vaccine. However, many mothers are unaware of these potential adverse events. Some mothers have even decided not to bring their children for vaccination due to concerns about side effects.” (HCW 2: Oromia)*

Similarly, the findings from the Somali region highlight that due to a lack of formal education and low awareness among the majority of the community, healthcare workers face challenges during vaccination services. Furthermore, a rumor circulates among certain areas of the community suggesting that the vaccine itself can cause illness. These factors act as barriers that negatively impact vaccination practices in the region.

Participants also mentioned that when the BCG and Measles vaccines are withheld from a few children to prevent vaccine wastage, it leads to disappointment among caregivers who expected their child to receive the vaccine during their visit.

*“As you know the BCG vaccine need twenty kids to be opened. For example, we won’t open the vaccine for five kids. This is because it will be wasted. They may go back feeling bad. These mothers may think not to come for next day appointment, due to transportation cost while the numbers of children may not be equivalent to the dose especially measles or BCG.” (HCW 2: Male, Gambella)*

However, community collaboration and engagement, nutritional support provided during vaccination campaigns, early announcement of schedules, and the involvement of community leaders are identified as beneficial facilitators for the vaccination service.

*“During the vaccination service, a nutritious food called FAFA is given to lactating mothers and pregnant women, and PLUMPY NUT is given to children. In this case, parents come to the health facility looking for these things, and we will provide them with the vaccination service there, which will help to avoid the interruption of vaccination.” (HCW 2: Afar)*

### Theme four: Vaccine acceptance

This theme includes the barriers and enablers to accept or refuse vaccine encountered during provision of routine immunization services at health facilities, or outreach/mobile program.

The results obtained from Addis Ababa city indicate that information spreads quickly within the community residing in slum areas. As a result, rumors pertaining to their vaccination experiences are shared among the community, which subsequently impacts vaccine acceptance within that community.

*“Residents of this village are more prone to gossips because there is high likelihood for the information to disseminate. Vaccine acceptance is low in such communities who live in slum areas. Especially if a side effect is seen on one child, it would be talked everywhere in the village. They exchange information among each other very fast. There are many unvaccinated children in this area. They may come for the first few vaccines and mightn’t come back for the next appointment if the child had developed a minor adverse effect. They don’t care that much.” (HCW1: Female, A.A)*

The finding from the Afar region indicated that vaccine acceptance during vaccine campaigns is high. This is attributed to the additional benefit of the campaigns, which provide an opportunity for their children to receive treatment for other health concerns as well.

*“The vaccine campaign has been highly accepted by the community. Some people have asked us to bring the vaccine again, even if they were not in the area during the vaccination campaign. The acceptance is high because it creates an opportunity to provide treatment and medicines to other children who are sick.” (HCW 2: Afar)*

Additionally, it is mentioned that healthcare workers actively involve community and religious leaders in community conversations and awareness creation activities. This approach aims to enhance vaccine acceptance within the community by leveraging the influence and trust that community and religious leaders hold.

Based on their experience with vaccine campaigns, participants reported that some caregivers may refuse to immunize their children during the vaccination campaign, asserting that the vaccine is toxic and could harm their children. In addition, some caregivers believe that the vaccines are being provided solely for the purpose of earning a salary or receiving incentives.

### Theme five: Health system factors

This theme summarizes the health system level factors which hinder/facilitate routine immunization service delivery across the regions.

### Vaccine supply related factors

Findings from Addis Ababa city shows an improvement in vaccine supply at health centers, although some areas continue to experience challenges due to an imbalance between population and vaccine availability. The utilization of multi-dose vials has proven to an effective strategy for reducing vaccine wastage; however, delays in administering BCG and measles vaccines can result in inefficiencies. In terms of service delivery and accessibility, immunization services are offered daily and free of charge in Addis Ababa and Oromia regions.

*“I think it would be better if the dose of vaccines such as BCG is reduced so that we can be able to give the service every day. In our setting, we wait for 2 weeks to open a BCG vial because it is prepared for 20 babies. The wastage rate is 50% which means we at least need 10 babies to open the vaccine. It could be difficult to get 10 babies in two weeks for some health centers. This hinders us from giving the vaccine as soon as a child is delivered” (EPI woreda focal: Addis Ababa)*

Similar to this, the finding from the Oromia regions showed that the vaccine supply is improved but in rare cases the number of vaccine request will not be matched with the number of clients. Security issues in Oromia region and transportation challenges in Afar hinder vaccine supply to certain areas.

Afar region also faces problems with power shortages and damaged refrigerators. Similarly, the Somali region experiences delays in reaching remote areas due to transportation limitations and lacks access to electricity, requiring more solar-powered refrigerators.

*“A challenge to our service is that there is a power shortage in our area and that the refrigerators we use for vaccination services in the health facility are damaged and not being repaired will hinder us from running the service as planned.” (EPI Focal, Afar)*

Overall, the use of multi-dose vials is a common factor contributing to challenges in vaccine supply across regions, causing inconvenience and dissatisfaction among caregivers.

### Managerial level factors

Relating with managerial level factors, the migration of people from different regions has led to an increased number of clients at health facilities, causing a shortage of vaccines in Addis Ababa city. This is because the estimation and delivery of vaccines are based on the population size in a given area. Additionally, there is a knowledge gap among managers regarding the understanding of the EPI program, and there is a low budget allocated for the EPI program at the sub-city level.

*“Top managers, CEOs, medical directors, and institution leaders don’t understand the importance of EPI as well as trained individuals. Of course, we are all health professionals, but those who have received training have a better understanding of it. Therefore, the top manager will assign one person for the vaccination service, assuming the patient flow is low. However, this will reduce both the quality and quantity of the service.” (Sub city EPI Focal: Addis Ababa)*

In the Afar region, a major challenge identified are the high staff turnover from the EPI program and poor leadership in addressing this turnover issue. Furthermore, the imbalanced distribution of the population in relation to the number of health centers during the restructuring of kebeles is recognized as a managerial factor in the region.

*“The challenges we encounter with health professionals primarily revolve around issues such as high attrition rates among trained vaccination personnel due to various reasons. Moreover, when new health professionals come, they often lack the necessary training in immunization practices, which affect their effectiveness in carrying out their duties.” (EPI Focal: Afar)*

Similarly, the Somali region also faces high staff turnover affecting the EPI program. Moreover, the finding highlights the reduction of immunization days to one day per week due to high workload, infrastructure problems, including remote areas lacking adequate facilities, insufficient funds for supportive supervision, and the unavailability of necessary materials such as refrigerators and monitoring charts, are also mentioned as managerial factors affecting the EPI program in the Somali region.

### Environmental level factors

Across the regions, insufficient transportation infrastructure, including a lack of motorcycles and vehicles, poses challenges for reaching remote areas and providing outreach services. Inadequate budget allocations and the absence of mobile health teams further hinder vaccination efforts.

Limited space in health centers, unavailability of refrigeration facilities, and security issues are common factors impeding the delivery and transportation of vaccines across the regions.

*“…..related to the geographical challenges, the community frequently relocates from one place to another, such as from one district to another, from Kebele to Kebele, and even to neighboring districts. Unfortunately, there are instances where vehicles are not permitted in the areas where the community moves due to issues with the roads and transportation infrastructure. This further complicates the vaccination efforts in reaching these mobile populations.” (RHB officer: Afar)*

Additionally, factors such as high staff turnover rates, malfunctioning cold chain systems, poor road conditions, and a deficiency of public transportation infrastructure are prevalent challenges. The nomadic lifestyle of certain communities, such as the Gambella and Somali communities, and their movement to hard-to-reach areas exacerbate these barriers. Furthermore, in the Gambella region, incomplete infrastructure and the seasonal impact of floods affecting road accessibility, leading to boat travel, contribute to low EPI coverage.

*“There is a lack of infrastructure, particularly roads, in the region, especially during the summer season when floods can inundate many areas. Akobo woreda, in particular, faces challenges with road accessibility, leading people to traveling by boat during this season. As a result, this situation contributes to low EPI coverage in the region.” (EPI Focal: Gambella)*

### Theme six: Health system best practices

This theme summarizes the health system policies or best practices which were effective in enhancing routine immunization service utilization across the regions.

Across different regions, community mobilization and engagement emerged as a common effective strategy for increasing the routine childhood immunization utilization. This included activities such as vaccine outreach, involvement of local organizations, NGOs, faith-based organizations, and community volunteers.

The finding from the Addis Ababa city indicated, there have been notable improvements in logistics and vaccine supply compared to previous times. The inclusion of the EPI program in private health institutions has also proven to be a valuable support for the immunization services. The findings from the Afar region indicated the implementation of a mobile health team for vaccination outreach programs and the establishment of a strong linkage system between local leaders and health workers were identified as successful strategies.

*“In order to enhance the accessibility of vaccination services to the community, the leaders and committees in the Kebele are notified in advance about the vaccination dates and locations through the health extensions and then they will inform the community to attend the designated vaccination site. Additionally, local leaders and committees play a vital role in immediately reporting any epidemic outbreaks in their locality to the district authorities. Therefore, they help us to facilitate the relationship with the community.” (Woreda EPI Focal: Afar)*

In Oromia region, engaging local organizations and faith-based groups played a significant role in enhancing routine immunization. Similarly, the findings from the Gambella region revealed that having community volunteers who actively mobilize the community for vaccination, while working in collaboration with health extension workers, was identified as a good practice in raising awareness, engaging community members, and promoting the importance of vaccination.

In the Somali region, engaging religious leaders and partners to create awareness about immunization, particularly during outbreaks, and the provision of routine health education by healthcare providers at the health centers were identified as best practices on immunization. The improvement in vaccine supply, in comparison to previous periods, was also highlighted as a good practice.

*“…there has been a notable growth in community mobilization, public conferences, and active community engagement. These efforts have resulted in a significant increase in vaccine uptake in the community.” (EPI Technical Assistant: Somali)*

### Theme seven: Participants’ Suggestions

This theme summarizes the suggestions given by the participants for improving immunization service in the regions. Finding from the Afar region have suggested strengthening the capacity and ensuring the necessary resources and materials are provided to health posts in hard-to-reach and remote areas. Additionally, they have suggested making health team leaders and healthcare providers accountable for monitoring the plan and reviewing the EPI program at the health facilities.

*“Due to the scattered living conditions of the Afar people, accessing health facilities for necessary healthcare services is challenging. To improve accessibility, it is recommended to provide monthly campaign services in the communities’ locations. Ensuring car access to vaccination services is crucial for effectively delivering healthcare services in hard-to-reach areas.” (KII 1: RHB Afar)*

The finding from Addis Ababa city highlights, the need for additional partners, community-based interventions, capacity building, and streamlining the vaccination service. The Oromia region finding emphasizes the importance of responsibility and accountability among all levels of health professionals. Additionally, it was recommended to provide additional support for mothers and children like nutritional support, as this can help increase utilization of the vaccination service.

The finding from the Gambella region indicated, strengthening the vaccine supply system, providing training for healthcare workers, and increasing manpower for the EPI program. The Participants from Somali region recommends implementing supportive supervision and mentoring programs for healthcare workers, providing on-the-job trainings, improving cold-chain management for proper vaccine storage and handling, strengthening review meetings and data quality checking systems, supporting mobile health teams to reach hard-to-reach areas, and improving vaccine supply for remote areas.

“*Delivering the vaccine is thus the challenge. Now, the vaccine maybe in Nageyle and there are three zones: Liban, Afder, Dawa access it from there. A small pickup car travels between these zones and it takes three months to deliver the vaccine to all of them. How would a small car deliver vaccine to three zones?*” (*KII1: Male RHB Somali)*

The recommendation from health care providers included improving the cold chain system and ensuring sufficient vaccine supply through transportation services across the regions. Additionally, creating awareness about the importance of vaccination, utilizing community groups for health education, and involving influential individuals in community mobilization activities are suggested strategies to enhance acceptance and coverage.

“*…people come to the health center in the early morning. They stay in the waiting area until they get their card. So it is good if “mass media” (teaching materials such as audiovisual) are available at this waiting area, and people learn from it about their health. This may change perception. They can raise questions and bring them to us*.” (HCW1: Oromia)

*“If healthcare providers are trained and motivated, their skills will be improved. Science is dynamic, and keeping healthcare providers up to date is crucial. When healthcare providers are kept up to date, they can also keep mothers informed by transferring their knowledge. Conversely, if healthcare providers are not up to date, the healthcare system may collapse, reverting to more traditional practices. Therefore, it is important to ensure that healthcare providers are properly motivated and provided with ongoing training and education.” (HCW 1: Somali)*

Based on the findings from the Addis Ababa city study, the key recommendations includes to enlist additional partners to provide financial support for the EPI program budget, sub-city should prioritize and dedicate efforts to reach the hard-to-reach areas within their respective catchment areas and enhancing capacity building activities for professionals involved in the program. Moreover, it is recommended that community-based interventions should be implemented to improve vaccine uptake by involving community leaders and influencers in the planning process. The participants also recommended to minimize the vaccine dose in order to streamline the vaccination service.

“*It was good if we had professional who took all mid-level training. In our city, there is only one girl who took mid-level EPI management (MLM) training. We want our professionals working in that area is qualified.*” (KII 3: Female EPI Focal Addis Ababa)

## Discussion

This study explored key health system factors affecting routine childhood immunization services in urban slum and pastoral regions of Ethiopia, collecting insights from health professionals across five regions including Addis Ababa city. The results explored a complex interplay between healthcare providers’ knowledge and attitudes, service delivery experiences, vaccine acceptance, and systemic infrastructure and management challenges.

Most healthcare providers explained their awareness of the importance of routine immunization in preventing vaccine-preventable diseases, which is consistent with previous findings that emphasize the central role of frontline health workers in vaccine advocacy [20–24].

Healthcare workers showed reduced motivation to deliver vaccination services mostly due to systemic challenges such as high workload, low incentives, infrastructure limitations, and inadequate training. This finding is in line with previous research; the lack of supportive work environments reduces motivation among healthcare workers and diminishes service quality [25–27]. However, according to findings from previous studies on health worker retention and performance in LMICs, intrinsic motivation like the satisfaction of helping children and community acknowledgment remained a significant driver [14,28].

Vaccine service delivery was particularly strained in pastoral regions where mobility, low literacy, and geographic dispersion hindered access to the service. These findings support the challenges highlighted by other studies in pastoralist and rural settings, where vaccine coverage is often lower due to logistical and supply difficulties [4,29–32]. However, the use of mobile health teams and community-based mobilization were reported as effective, reinforcing WHO’s recommendation for adaptive service delivery models in hard-to-reach populations [11]. These strategies are particularly effective in Ethiopia because they are tailored to the mobility of pastoralist communities, reducing geographical barriers to access health services and improves equity among underserved populations.

Rumors and misconceptions about vaccines in general were major community-level barriers. This aligns with evidence from other study suggesting that misinformation and fear of adverse events can significantly affect vaccine acceptance [7,33–35]. In these contexts, involving trusted community members such as religious leaders, women’s groups, and local officials proved to be effective in increasing vaccine acceptance [36,37].

Health system constraints, including irregular vaccine supply, multi-dose vials of the vaccine, cold chain interruptions, and poor transport infrastructure, negatively affected immunization services. These structural inadequacies are consistent with findings from health systems research across sub-Saharan Africa, where supply chain inefficiencies and weak cold chain logistics are recurring problems [31,38]. To enhance vaccine coverage and regular provision of the vaccination services, the need for improving and solving health system barriers related to logistics and supply of vaccines is highly prioritized.

Managerially related challenges like insufficient EPI prioritization, staff turnover, and lack of budgetary support were frequently mentioned in this study. These findings reflect broader systemic gaps in health workforce planning and program implementation that affect immunization program performance [39]. Addressing these governance-related challenges will require health sector commitment, adequate budgeting, and greater investment in EPI-specific leadership.

Despite these health system-related challenges, notable best practices were identified. Community mobilization, integration of private health facilities, mobile outreach teams, and coordination with kebele leaders significantly improved vaccine uptake. This is aligned with global best practices that emphasize integrated service delivery, community engagement, and public-private partnerships to enhance immunization access [40]. Furthermore, providing nutritional services like during immunization campaigns effectively boosted vaccine uptake, suggesting that combining with other health services can enhance program reach.

### Policy and practice implications of the study

Unlike previous studies that primarily focus on barriers to immunization, our study also identified context-specific opportunities, including the successful integration of private health facilities, nutritional supplements as positive reinforcement, and community-led mobilization to improve vaccine uptake in marginalized settings. These findings suggest that to address health system constraints of immunization services delivery, scaling up locally tailored service delivery approaches that respond to the distinct needs of urban slum and pastoralist communities is mandatory.

National and regional immunization programs should prioritize equitable resource allocation, strengthen vaccine supply chains, expand and repair existing cold chain facilities, ensure adequate human power, give regular in-service training, provide supportive supervision, and incentives for frontline health workers. Policymakers should also consider contextual service delivery models, including mobile outreach services and flexible vaccination schedules, to improve access among mobile pastoralist populations. Furthermore, integrating private health facilities and multisectoral collaboration with local community structures and trusted bodies can enhance immunization service coverage in these communities.

### Strengths and limitation of the study

The inclusion of marginalized settings and diverse regions of urban slums and pastoralist areas, enaging multistakeholders (healthcare workers, HEWs, program managers) is one of the major stregnths of this study. Use of multiple frameworks such as HCD and BeSD to guide data collection and analysis is the other strength of this study demonstrating its methodological rigor and contextual relevance.

However, this study is not without limitation. The study findings are based on interviews with health system stakeholders and therefore do not reflect the perspectives of caregivers or community members, which may limit the understanding of demand-side factors influencing routine childhood vaccination service delivery. Future studies incorporating both health system and community perspectives would provide a more comprehensive understanding of the factors influencing routine childhood immunization.

## Conclusion

Overall, although some regions experienced improvements in vaccine availability, significant challenges persist. These include inconsistent vaccine supply, limited cold-chain capacity, low financing, high workloads, challenges in reaching these populations, mismatches between population size and vaccine supply, and inefficient use of multi-dose vials. Together, these health system gaps require deliberate attention and targeted actions to strengthen vaccination services across regions.

At the same time, strong community mobilization and engagement emerged as effective practices for improving immunization services. Expanding EPI services to private health facilities, deploying mobile health teams, and reinforcing coordination between kebele leaders and health workers proved successful and should be further scaled to reach under-immunized populations.

Enhancing vaccine coverage and equity in Ethiopia will require focused policy reforms that tackle both demand- and supply-side constraints. On the demand side, greater investment in culturally responsive education, engagement of trusted community actors, and sustained community participation is essential. On the supply side, closing cold chain gaps, providing regular training, strengthening vaccine financing, improving working conditions, and upgrading logistics systems could substantially improve the delivery of routine immunization services. Overall, by strengthening health system capacity while fostering meaningful community involvement, Ethiopia can move closer to comprehensive vaccine coverage and more effectively reach under-immunized communities.

## Supporting information

S1 File Code Book For KII and HCWs. (DOCX)

S2 File PLOS_Human_Participants_Research_Checklist_2025. (PDF)

## Abbreviations

BeSD: Behavioral and Social Drivers
EPI: Expanded Program on Immunization
HCD: Human-Centered Design
HCW: Healthcare Workers
HEW: Health Extension Worker
KII: Key Informant Interview
RMNCH: Reproductive, Maternal, Newborn, and Child Health
