# Supplementary material for: Challenges, opportunities and best practices in routine childhood vaccination services delivery in urban slums and pastoralist regions of Ethiopia: An exploratory qualitative study in marginalized settings
Source: PLoS One. 2026 Aug 21;21(8):e0356253. doi: 10.1371/journal.pone.0356253 (PMC13497257; doi:10.1371/journal.pone.0356253)
Supplement: Supplementary file 2 [file pone.0356253.s001.docx]

**Codebook for Health care Workers (HCWs)**

**Title of the research project:** Routine Childhood Vaccination Services Delivery in Urban Slums and Pastoralist Regions of Ethiopia: An Exploratory Qualitative Study in marginalized settings.

**Objectives:**

1. To identify health system and environmental level barriers and enablers of vaccination among the selected facilities in communities with high number of zero-dose children.

| **Theme** | **code** | **Description** |
| --- | --- | --- |
| **Knowledge and attitude** | - Mind Mapping | Assesses the health care providers knowledge, mechanisms of upgrading their knowledge and skills, beliefs about routine immunization and COVID-19 vaccine, MNCH services and the service users |
| **Task and division of labor** | - Services provided | This includes the number of days and frequency per week/per month the HCWs are involved in providing immunization services. Separately, also explore the time and frequency they spent for immunization related counseling, health education and community mobilization. It also includes for whom they are providing those services. |
| **Intention** | - Barriers | Includes factors that reduce the intention of health care providers to give immunization services example can be, transportation, low or per diem, poor governance and leadership etc. |
|  | - Enablers | Includes factors that enhance the intention of health care providers to give immunization services. Example: trainings, incentives, career development, promotion, recognition … etc. |
| **Immunization service** | - Vaccination service experience | This includes the barriers and facilitators encountered by healthcare providers when delivering routine immunization and COVID-19 vaccination services, whether at the health facility, through house-to-house visits, or at outreach and mobile sites. |
| **Vaccine Acceptance** | - acceptance /confidence | This includes the barriers and enablers faced during provision of routine immunization at health facility, house to house or outreach/mobile program related with the vaccine acceptance it also includes any challenges faced during the immunization service delivery related with vaccine acceptance both for COVID-19 and routine immunization. |
| **Recommendations** | Suggestion to improve immunization service by HCWs | The suggestions given by the health care providers to enhance routine childhood immunization services in their community. This includes two most common recommendations mentioned by the participants regarding immunization. |

**Codebook for KII**

**Title of the research project:** Routine Childhood Vaccination Services Delivery in Urban Slums and Pastoralist Regions of Ethiopia: An Exploratory Qualitative Study in marginalized settings.

**Objectives:**

1. To identify health system and environmental level barriers and enablers of vaccination among the selected facilities in communities with high number of zero-dose children.

| **Theme** | **code** | **Description** |
| --- | --- | --- |
| **Intention** | - Motivation/satisfaction | Includes factors affecting health professionals intention to provide immunization services which can either motivate or demotivate HCWs example: capacity building trainings, policies and strategies. |
| **health system factors** | - vaccine supply related factors | Includes system level vaccine supply related factors which hinder/facilitate routine immunization service delivery. Example: like availability, accessibility, delivery, vaccine wastage concern, service convenience like service provision days in a week and hour in a day  etc. |
|  | - managerial factors | This includes leadership, administrative, governance, managerial and human resource related factors which can either facilitate or hinder routine immunization service provision. |
|  | - environmental-level factors | This includes geographical or environmental level factors which included infrastructure, logistics, and transportation that can either facilitate or hinder immunization service provision. |
| **health system best practices** | - Best practices | Health system policies or best practices which were effective in enhancing routine immunization service utilization example: Community participation in the immunization session preparation, community’s ownership and engagement on immunization services including community dialogue sessions /HCD approach etc. |
| **community context** | - Socio-cultural context | Involves socio-cultural issues related with immunization which can either enable or inhibit routine immunization. for example: beliefs, norms, values, cultural practices etc. |
|  | - Community structure /platforms | Includes availability and list of community platforms/networks, mobile nature of the community and that promote routine immunization. |
|  | - community-level interventions | Community level public health interventions, strategies, best practices and initiatives used to enhance routine immunization service utilization. This also includes interventions to track the community mobility and discontinuation of vaccinations among the community. |
| **Data Quality** | - data reporting system | This includes the documentation, storage, reporting and utilization of immunization data including community mobilization activities. It also includes the how the quality data is ensured. |
|  | - service integration and continuity of care | This includes the integration of different health services within the health facility. For example the linkage of maternal health services, nutrition or WASH with immunization services |
| **Financing mechanism** | - stakeholders engagement | This includes key stakeholders who support immunization, the type of support given (financially and/or technically) and the impact of this stakeholder collaboration on immunization service utilization. |
| **Recommendations** | - the way forward by the KII’s | The suggestions given by the key informants/participants to improve immunization service in the region/area. |
